# Growth arrest-specific protein 6 plasma concentrations during septic shock

**DOI:** 10.1186/cc5158

**Published:** 2007-01-22

**Authors:** Sébastien Gibot, Frédéric Massin, Aurélie Cravoisy, Rachel Dupays, Damien Barraud, Lionel Nace, Pierre-Edouard Bollaert

**Affiliations:** 1 Service de Réanimation Médicale, Avenue du Maréchal de Lattre de Tassigny, Hôpital Central, Nancy, 54000, France; 2 Projet Avenir INSERM, Coordination Circulation; 3 Laboratoire d'Immunologie, Avenue de la Foret de Haye, Faculté de Médecine, Nancy, 54000, France

## Abstract

**Introduction:**

The product of growth arrest-specific gene 6 (Gas6) is a vitamin K dependent protein that is secreted by leucocytes and endothelial cells in response to injury and participates in cell survival, proliferation, migration and adhesion. Our purpose was to investigate plasma Gas6 concentration and its relation to organ dysfunction in patients with septic shock.

**Methods:**

Forty-five patients with septic shock admitted to a medical adult intensive care unit were enrolled. Plasma Gas6 concentration was determined using enzyme-linked immunosorbent assay at days 1, 3, 7 and 14.

**Results:**

The median (interquartile range) Gas6 concentration was 51 (5 to 95) pg/ml at admission. A positive correlation (Spearman rank-order coefficient [rs] = 0.37, *P *= 0.01) was found between Gas6 level and Sepsis-related Organ Failure Assessment score. Patients requiring renal support had higher Gas6 concentration that those without need for haemofiltration (76.5 [52 to 164] pg/ml versus 10.5 [1.5 to 80.5] pg/ml; *P *= 0.04). Moreover, there was a positive correlation between Gas6 and aspartate transaminase (rs = 0.42, *P *= 0.006) and between Gas6 and prothrombin time (rs = 0.45, *P *= 0.02). Although there was a progressive decline in Gas6 concentration in survivors (analysis of variance, *P *= 0.01), nonsurvivors exhibited persistently elevated Gas6. However, the two populations diverged only after day 7 (*P *= 0.04).

**Conclusion:**

Plasma concentrations of Gas6 correlate with disease severity, especially with renal and hepatic dysfunction, in septic shock.

## Introduction

As new therapeutic options emerge for the management of septic shock, accurate prognostic factors are needed to better identify those patients who are likely to benefit. Although a number of severity scores (including Acute Physiology and Chronic Health Evaluation II score and Simplified Acute Physiology Score [SAPS]II or SAPSIII) and biological markers (procalcitonin [PCT] and C-reactive protein, among others) are available to predict outcome in critically ill patients, the appropriateness of their use during septic shock remains debatable.

The product of growth arrest-specific gene 6 (Gas6) recently attracted attention because it was found to be elevated during sepsis and may correlate with organ dysfunction [1]. This vitamin K dependent protein is secreted by leucocytes and endothelial cells in response to serum starvation or injury, and is the biological ligand for the Axl subfamily of receptor tyrosine kinases comprising Axl, Sky and Mer [2]. The Gas6/Axl system participates in cell survival [3,4], proliferation [5], migration [6] and adhesion [7]. Gas6 is also thought to act as a recognition bridge between apoptotic cells and phagocytes that ingest them [8].

Evidence of a role for the Gas6/Axl system during sepsis has been obtained in mice. Camenisch and coworkers [9] observed that mice that lack the intracellular domain of c-mer exhibited increased lipopolysaccharide (LPS)-induced tumor necrosis factor-α production and suffered increased mortality after LPS administration *in vivo*. Moreover, the same group demonstrated that mice deficient in c-mer had impaired clearance of apoptotic cells [10].

These data prompted us to investigate plasma concentrations of Gas6 during septic shock and its relationship with severity and outcome.

## Materials and methods

### Study population

Between August 2005 and February 2006, all consecutive patients admitted with septic shock into a 16-bed medical intensive care unit of a teaching hospital were enrolled. The diagnosis of septic shock was established on the basis of current definitions [11]. Patients were not enrolled if they were older than 80 years or were immunocompromised (treatment with corticosteroids > 1 mg/kg prednisone or equivalent, bone marrow or organ transplant recipients, neutropenia < 0.5 10^9^/l, haematologic malignancy, or AIDS). The institutional review board granted approval, and informed consent was obtained from patients or their relatives before their inclusion.

### Data collection

Upon admission to the intensive care unit, the following items were recorded: age, sex, severity of underlying medical condition stratified according to the criteria of McCabe and Jackson, SAPSII, Sepsis-related Organ Failure Assessment (SOFA) score, vital signs, respiratory variables, routine blood tests and microbial culture results. Outcome was assessed over a 28-day follow-up period.

### Measurement of sTREM-1 and Gas6 plasma concentrations

Day 1 was defined as the day of admission to the intensive care unit. Within 12 hours after admission and enrolment in the study, 5 ml whole heparinized blood was drawn via an arterial catheter for determination of soluble triggering receptor expressed on myeloid cells-(sTREM)-1 and Gas6 determinations. Assessment of plasma sTREM-1 levels was performed as described elsewhere [12]. For Gas6 determination, microtitre plates were coated overnight at room temperature with 4 μg/ml of polyclonal mouse anti-human Gas6 antibody (RnD Systems, Lille, France). After three washes with 0.05% Tween 20 in phosphate-buffered saline, wells were blocked with 1% bovine serum albumin in phosphate-buffered saline for one hour at room temperature. Three additional washes were then performed and 100 μl plasma or standards (recombinant human Gas6; RnD Systems) were added for two hours at room temperature. Washes were repeated and 100 ng/ml biotinylated monoclonal goat anti-human Gas6 antibody (RnD Systems) added for two hours at room temperature. Detection was performed with peroxydase-conjugated streptavidin. Measurements were repeated three times, and intra-assay and inter-assay coefficients of variation were 6.3% and 7.8%, respectively.

Repeated determinations of sTREM-1 and Gas6 plasma concentrations were performed on days 1, 3, 7 and 14.

### Statistical analysis

Descriptive results of continuous variables are expressed as mean ± standard deviation. Non-normally distributed values, as assessed using the Kolmogorov-Smirnov test, are reported as median (interquartile range). Correlations between Gas6 plasma concentration and clinical or biological parameters were investigated using the Spearman test. Gas6 was also tested for its association with several variables using the Mann-Whitney *U *test. The time course of Gas6 plasma level was assessed using analysis of variance. Analyses were completed using Statview software (Abacus Concepts, Berkeley, CA, USA), and two-tailed *P *< 0.05 was deemed statistically significant.

## Results

### Characteristics of the studied population

Forty-five septic shock patients were included. They were seriously ill, as indicated by need for mechanical ventilation and vasopressors in all. The main clinical and biological characteristics are summarized in Table 1. Infection originated from the lung (community-acquired pneumonia; 62%), the urinary tract (pyolonephritis; 15%), the abdomen (peritonitis; 11%), the heart (endocarditis; 4%) or the skin (necrotizing fasciitis; 4%). The aetiology remained unknown in two patients. Microbiological documentation was obtained in 31 (68.9%) patients (Table 2). The high 28-day mortality rate (46.6%) was to be expected, based on the elevated SAPSII and SOFA scores.

**Table 1:** Characteristics of patients at inclusion

Characteristic/parameter	Patients with septic shock (*n *= 45)
Male (*n *[%])	31 (69)
Age (years; mean ± SD)	62.3 ± 17.7
SAPSII score (mean ± SD)	64.4 ± 19.3
SOFA score (median [IQR])	12.5 (11.5–15)
Vasopressors (*n *[%])	45 (100)
Mechanical ventilation (*n *[%])	45 (100)
CVVH (*n *[%])	12 (26.6)
Corticosteroids (*n *[%])	36 (80)
Drotrecogin alfa (activated; *n *[%])	7 (15.5)
Pao_2_/Fio_2 _(median [IQR])	150 (100–180)
Lactate (mmol/l; median [IQR])	2.9 (1.9–4.5)
C-reactive protein (mg/l; median [IQR])	157 (95–225)
Procalcitonin (ng/ml; median [IQR])	12.6 (3.5–49.6)
sTREM-1 (pg/ml; median [IQR])	117.5 (37–219.5)
Gas6 (pg/ml; median [IQR])	51 (5–95)
Mortality rate (*n *[%])	21 (46.6)

CVVH, continuous venovenous haemofiltration; Fio_2_, fractional inspired oxygen; Gas6, growth arrest-specific gene 6 product; IQR, interquartile range; Pao_2_, arterial oxygen tension; SAPS, Simplified Acute Physiology Score; SD, standard deviation; SOFA, Sepsis-related Organ Failure Assessment; sTREM, soluble triggering receptor expressed on myeloid cells.

**Table 2:** Micro-organisms associated with sepsis

Micro-organisms	Patients with septic shock (*n *= 45)
Bacilli	
*Escherichia coli*	11 (24.4)
*Haemophilus *spp.	1 (2.2)
*Pseudomonas aeruginosa*	1 (2.2)
*Stenotrophomonas maltophilia*	1 (2.2)
Cocci	
*Streptococcus *spp.	13 (28.8)
MSSA	3 (6.6)
*Enterococcus *spp.	1 (2.2)
Unknown	14 (31.1)

Values are expressed as the number (%) of micro-organisms isolated. Because of rounding, percentages do not add up to 100. MSSA, methicilin-sensitive *Staphylococcus aureus*.

### Baseline plasma concentration of Gas6

**Figure 1:**
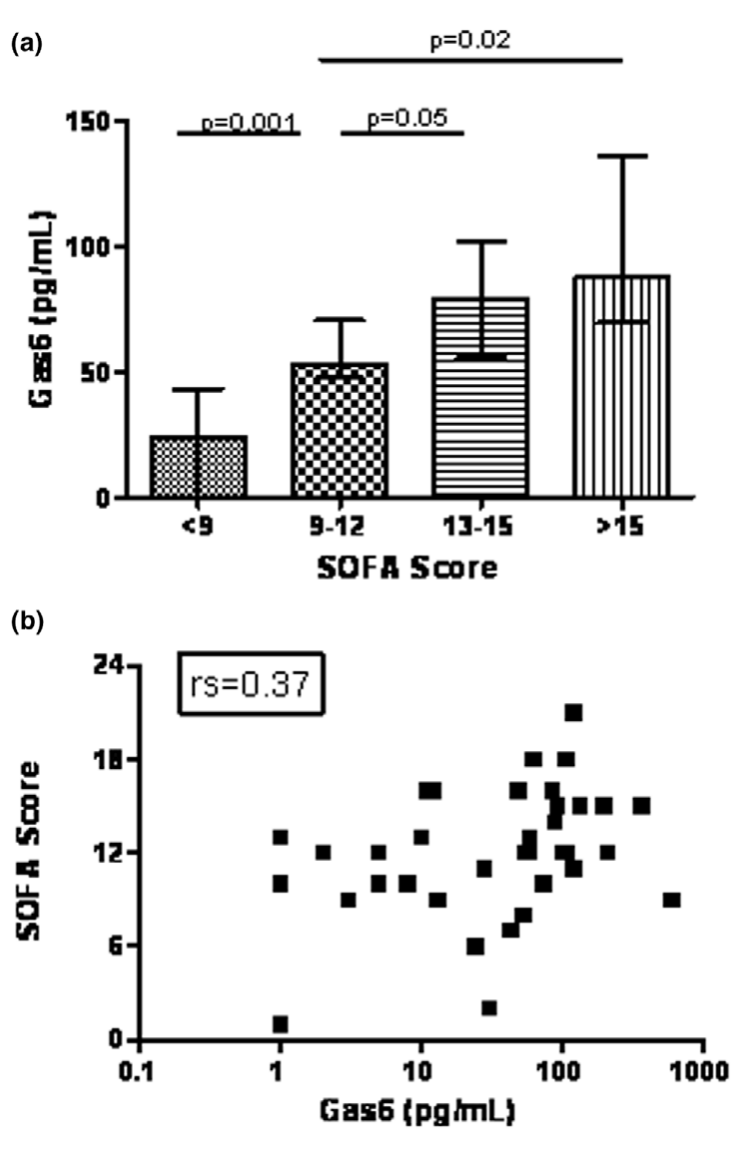
Correlation between plasma Gas6 concentration and SOFA score. **(a) **SOFA scores were separated into quartiles and plotted against Gas6 plasma concentration. Groups were compared using the Mann-Whitney *U *test. **(b) **Individual SOFA scores were plotted against Gas6 concentration and the correlation was evaluated using the Spearman test (rs = 0.37, *P *= 0.01). Gas6, growth arrest-specific gene 6 product; SOFA, Sepsis-related Organ Failure Assessment.

**Figure 2:**
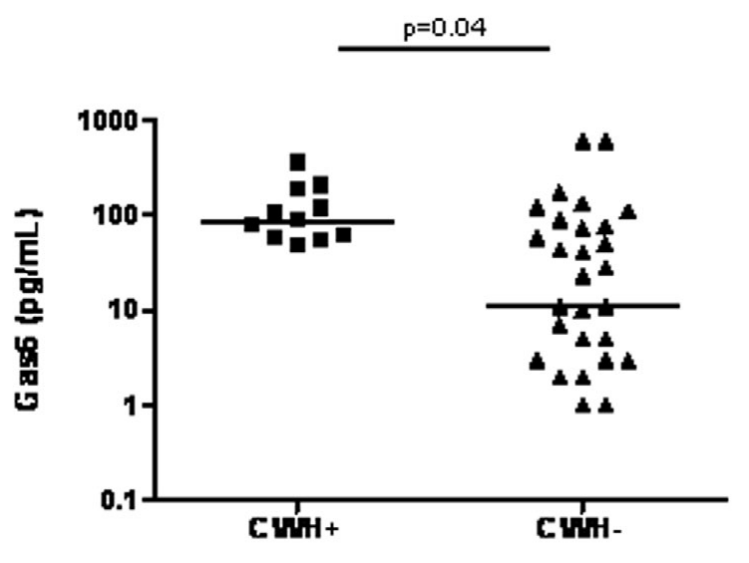
Plasma Gas6 concentrations according to need for CVVH. The two groups were compared using the Mann-Whitney *U *test. CVVH, continuous venovenous haemofiltration; Gas6, growth arrest-specific gene 6 product.

Median (interquartile range) Gas6 concentration was 51 (5 to 95) pg/ml at admission. Although Gas6 level did not differ between survivors and nonsurvivors, a positive correlation (Spearman rank-order coefficient [rs] = 0.37, *P *= 0.01) was observed with SOFA score (Figure 1). However, none of the components of the SOFA score taken individually (arterial oxygen tension/fractional inspired oxygen, platelet count, bilirubinaemia, mean arterial pressure, creatininaemia and Glasgow Coma Scale score) correlated with Gas6 concentration. Within the first 24 hours after admission, 12 patients required renal support (continuous venovenous haemofiltration) because of acute renal failure. We observed higher Gas6 concentrations among these patients than in those without need for renal support (76.5 [52 to 164] pg/ml versus 10.5 [1.5 to 80.5] pg/ml; *P *= 0.04; Figure 2). Gas6 was also related to hepatic function; there were positive correlations between Gas6 and aspartate transaminase (rs = 0.42, *P *= 0.006) and between Gas6 and prothrombin time PT (rs = 0.45, *P *= 0.02). Finally, Gas6 was strongly correlated with plasma concentrations of PCT (rs = 0.44, *P *= 0.005) and sTREM-1 (rs = 0.45, *P *= 0.003). No relation between Gas6 and other clinical or biological features (age, sex, source of infection, infecting micro-organism, lactate, leucocyte count, and so on) was observed.

### Time course of Gas6 plasma concentration

**Figure 3:**
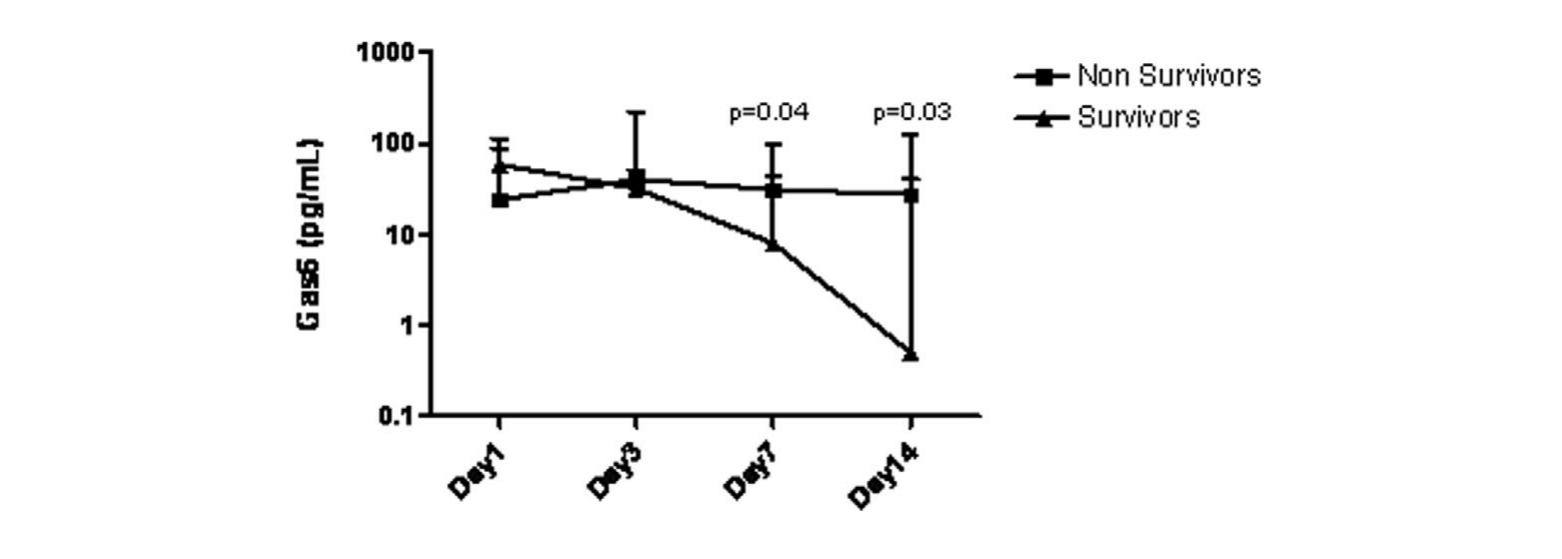
Time course of plasma Gas6 in surviving versus nonsurviving patients. Values are expressed as median with interquartile range. Gas6, growth arrest-specific gene 6 product.

Although there was a progressive decline in Gas6 concentration in the survivors (analysis of variance, *P *= 0.01), nonsurvivors exhibited persistently elevated Gas6 level (Figure 3). However, the two populations diverged only after day 7 (*P *= 0.04), and neither the day 7 Gas6 concentration nor the difference between days 7 and 1 were sufficiently accurate to predict final outcome (area under the receiver operating characteristic curves: 0.58 [0.48 to 0.68] and 0.62 [0.50 to 0.74], respectively; *P *> 0.05).

## Discussion

The main finding of this study is that Gas6 plasma concentration correlates with organ dysfunction, especially that of kidney and liver, and several markers of infection during septic shock.

The product of growth arrest-specific gene 6, Gas6, is the biological ligand for the receptor tyrosine kinase Axl and is implicated in cell survival, proliferation, migration, adhesion and recognition of dying cells [3-8]. Moreover, the Axl system has been shown to be essential for functional maturation of natural killer cells and normal expression of inhibitory and activating natural killer cell receptors [13]. On one hand, Gas6 thus appears to be of crucial importance in maintaining cell function. Evidence to support this view comes from an experimental model of endotoxaemia [9], in which mice lacking the intracellular domain of c-mer exhibited increased LPS-induced tumour necrosis factor-α production and suffered increased mortality after LPS administration *in vivo*. However, in support of findings recently reported by Borgel and coworkers [1], we observed a good correlation between Gas6 concentration and organ dysfunction during septic shock. How can these findings be reconciled?

First, because Gas6, via Akt, causes an increase in the antiapoptotic protein Bcl-2 [14], it is tempting to link the delayed neutrophil apoptosis observed during sepsis [15] with the concentration of this protein. An extended neutrophil life time could therefore contribute to organ injury. Although Gas6 has clearly been shown to delay cell death both *in vitro *and *in vivo*, this explanation remains hypothetical because we did not specifically investigate apoptosis.

Second, a deleterious role for Gas6 has been established in kidney and hepatic disease. Increased glomerular expression of Gas6 has been detected in animal models of kidney disease [16], and Gas6 knockout mice were shown to be resistant to accelerated nephrotoxic nephritis [17]. Gas6 upregulation was also observed during allograft rejection in a rat kidney transplant rejection model [18] as well as in dysfunctional human renal allografts [19]. The link between Gas6 and renal injury is indirectly supported by our finding that patients requiring renal support exhbiited increased Gas6 plasma concentrations compared with those who did not need such support. Gas6 has also been implicated during hepatic injury [20], and we observed a correlation between Gas6 concentrations and hepatic dysfunction. Of course, it is not possible to determine here whether Gas6 is a bystander during renal and hepatic injury or directly contributes to dysfunction of these organs.

The finding that Gas6 strongly correlated with both PCT and sTREM-1 concentrations is difficult to explain. Gas6 has never shown to be a marker of infection, and neither PCT nor sTREM-1 are thought to be reliable severity markers during septic shock. Does Gas6 stimulate the release of these proteins? Although Gas6 has been shown *in vitro *to stimulate nuclear factor-κB binding activity and subsequent transcriptional activation from nuclear factor-κB responsive promoters [14,21], this hypothesis remains to be investigated.

Because Gas6 correlated well with organ dysfunction, we sought to evaluate whether it could be used as a marker of prognosis. Admission levels of Gas6 did not differ between survivors and nonsurvivors, and this finding is in accordance with that reported by Borgel and coworkers [1]. When patients were segregated according to outcome, we observed a divergent time course of Gas6 concentrations only at day 7 and after, and therefore Gas6 did not appear to be useful in predicting outcome during the early period of septic shock. Nevertheless, because Gas6 concentration was clearly linked to the degree of organ injury, its use as part of a panel of severity markers is interesting and could be further tested.

## Conclusion

Gas6 plasma concentrations correlate with severity, and especially renal and hepatic dysfunction, in septic shock. However, Gas6 by itself is not a reliable early indicator of outcome.

## Key messages

• Gas6 plasma concentrations correlate with organ injury during septic shock, especially hepatic and renal dysfunction.

• Neither admission level nor time course of Gas6 concentration are sufficiently discriminative to permit prediction of outcome during the early period of septic shock.

## Abbreviations

Gas6 = growth arrest-specific gene 6 product; LPS = lipopolysaccharide; PCT = procalcitonin; rs = Spearman rank-order coefficient; SOFA = Sepsis-related Organ Failure Assessment; sTREM = soluble triggering receptor expressed on myeloid cells.

## Competing interests

The authors declare that they have no competing interests.

## Authors' contributions

SG designed the study, enrolled patients, performed measurements and drafted the manuscript. FM performed measurements. AC, RD, DB and LN enrolled patients. PEB designed the study and drafted the manuscript. All authors approved the final version of the manuscript.
